# Classification of Motor Imagery Electroencephalography Signals Based on Image Processing Method

**DOI:** 10.3390/s21144646

**Published:** 2021-07-07

**Authors:** Zhongye Chen, Yijun Wang, Zhongyan Song

**Affiliations:** School of Electronic and Information Engineering, Changchun University of Science and Technology, Changchun 130022, China; 2019100469@mails.cust.edu.cn (Z.C.); 2019100474@mails.cust.edu.cn (Z.S.)

**Keywords:** brain-computer interface, motor imagery (MI), convolutional neural network (CNN), feature enhancement, attention module

## Abstract

In recent years, more and more frameworks have been applied to brain-computer interface technology, and electroencephalogram-based motor imagery (MI-EEG) is developing rapidly. However, it is still a challenge to improve the accuracy of MI-EEG classification. A deep learning framework termed IS-CBAM-convolutional neural network (CNN) is proposed to address the non-stationary nature, the temporal localization of excitation occurrence, and the frequency band distribution characteristics of the MI-EEG signal in this paper. First, according to the logically symmetrical relationship between the C3 and C4 channels, the result of the time-frequency image subtraction (IS) for the MI-EEG signal is used as the input of the classifier. It both reduces the redundancy and increases the feature differences of the input data. Second, the attention module is added to the classifier. A convolutional neural network is built as the base classifier, and information on the temporal location and frequency distribution of MI-EEG signal occurrences are adaptively extracted by introducing the Convolutional Block Attention Module (CBAM). This approach reduces irrelevant noise interference while increasing the robustness of the pattern. The performance of the framework was evaluated on BCI competition IV dataset 2b, where the mean accuracy reached 79.6%, and the average kappa value reached 0.592. The experimental results validate the feasibility of the framework and show the performance improvement of MI-EEG signal classification.

## 1. Introduction

The brain is the highest level part of the nervous system, and various functions of the human body have corresponding mapping areas on the brain, such as sensory areas and motor areas [1]. A brain-computer interface system is a communication system that enables the brain to interact with the outside world by connecting it to external devices [2]. Modern brain neuroscience has demonstrated that different changes in a person’s state of mind, emotions, and thoughts can affect changes in EEG signals, so it is feasible to study changes in a person’s mind through EEG signal analysis [3].

In recent years, brain signal research, such as steady-state visual evoked potentials, P300 evoked potentials, and motor imagery has also made great progress [4,5]. In this paper, MI-EEG will be further studied and discussed. The physiological basis of motor imagery is that when a person imagines movement of different parts of the body (such as left or right hands) without actually moving them, they also activate different functional areas of the brain accordingly, thus generating EEG signals with different properties. For example, when a person performs unilateral limb imagery movements, the μ and β rhythm energy in the ipsilateral sensorimotor cortex of the brain increases, and the contralateral μ and β rhythm energy decreases. This phenomenon is known as event-related synchronization and event-related desynchronization (ERD/ERS) [6,7].

The processing of motor imagery EEG signals generally includes signal acquisition, signal pre-processing, feature extraction, and classification recognition. Feature extraction is the most critical step among them. Currently, in feature extraction, the common spatial pattern (CSP) [8] is one of the effective methods. It uses matrices diagonalization of matrices to find an optimal set of spatial filters for projection, which maximizes the difference in variance values of different signals, resulting in a feature vector with a high degree of discrimination. However, CSP excessively relies on bandwidth selection. The filter bank common space model proposed by Zhang et al. solves this problem [9]. This method selects the most discriminative features by calculating the mutual information of CSP features from multiple sub-bands, but the extraction of the features is more complicated. To simplify the feature extraction operation, Tabar et al. convert the EEG signals of each channel into time-frequency images, which are classified by a deep network stacked autoencoder (SAE) [10]. The method simplifies the feature extraction step, but the classification performance needs to be improved. Zhang et al. feed the classifier with a combination of time-frequency images of multiple channels arranged up and down, and the classification accuracy is improved [11]. However, the time-frequency images arranged up and down contain too many irrelevant frequency bands and introduce a lot of noise.

In feature recognition, various deep learning models have also been applied to the feature recognition and classification of EEG signals. Liu et al. propose a classification framework for long short-term memory (LSTM) networks combined with channel weighting techniques, which has a small parameter size and faster processing speed [12]. Yang et al. rely on the basic framework of convolutional neural networks (CNNs) to construct an end-to-end classification model and introduce stacked sparse autoencoders to enhance the generalization ability of the model [13]. Considering the inconsistency and the possibility of distortion of the measured EEG signals, Ha et al. propose a classification model based on a capsule network to improve the classification ability of signals [14].

In this paper, we have improved the model in terms of enhancing the distinctness of feature differences in the input data and the classification model. An IS-CBAM-CNN deep learning framework is proposed from the perspective of image processing. Based on the logically symmetrical relationship between ERD/ERS and the C3 and C4 channels, a method based on image subtraction (IS) is proposed to enhance the feature representation of MI-EEG signals. The method obtains the time-frequency image of the signal by wavelet transform and then uses the result of the time-frequency image subtraction as the input to the classifier. A convolutional neural network with two convolutional layers and two pooling layers is built as the base classifier. Based on the temporal and frequency characteristics of the motor imagery EEG signal, the CBAM is added to convolutional neural networks to capture salient features of MI-EEG signals on images and enhance classifier recognition. The performance of the framework is evaluated on public datasets.

The rest of the paper is organized as follows: Section 2 reviews two datasets and noise processing. Section 3 describes an improved method in detail. The experimental results and discussion are presented in Section 4. Finally, we make our conclusions in Section 5.

## 2. Materials

### 2.1. Datasets

We used two public datasets to evaluate our model. The first dataset is BCI Competition IV dataset 2b [15]. The dataset consists of nine subjects from EEG data. For each subject, five sessions were provided. The first two contained training data without feedback and the last were recorded with feedback. In this paper, only data without feedback are selected as the dataset for each subject.

**Figure 1:**
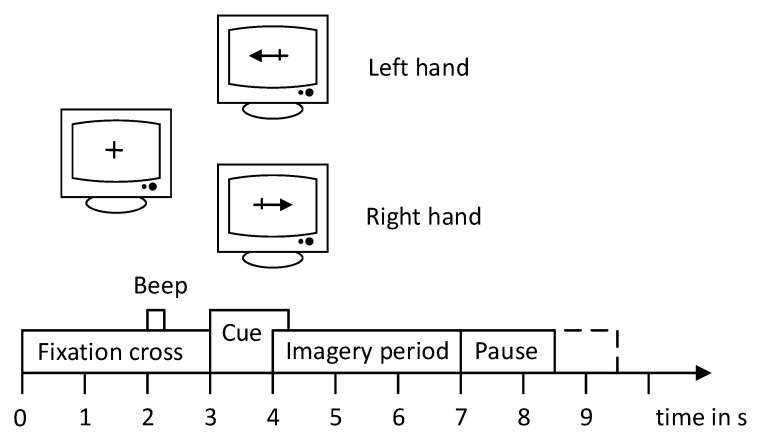
The paradigm of BCI competition IV 2b.

Each trial started with a fixed cross and an additional short audible warning tone (1 kHz, 70 ms). After a few seconds, a visual cue (with an arrow pointing to the left or right, depending on the category requested) appeared for 1.25 s. Subjects had to visualize the corresponding hand movements within 4 s. Each trial was followed by a short break of at least 1.5 s. In addition, the rest period was increased by up to 1 s of randomization to avoid adaptation. The experimental paradigm is the same for each experiment, as shown in Figure 1.

The dataset was recorded with three bipolar recordings (C3, Cz, and C4) at 250 Hz sampling frequency. They were band-pass filtered between 0.5 Hz and 100 Hz, with a 50 Hz trap filter enabled. The locations of the three bipolar recordings were slightly different for each subject. The data set included experiments on the motor imagery task for both right-handed and left-handed movements. Each session contained 120 trials.

**Figure 2:**
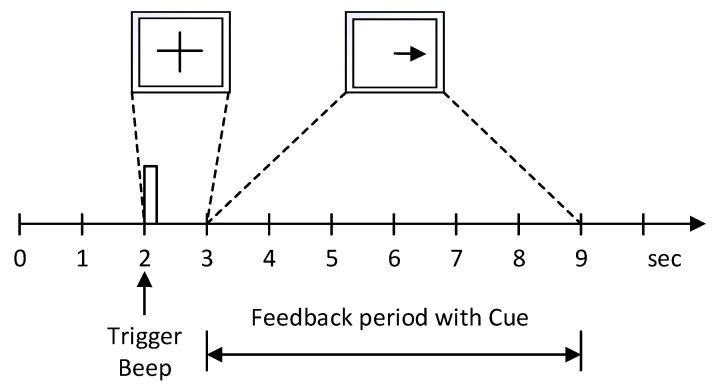
The paradigm of BCI competition II dataset III.

The second dataset used in this paper is BCI Competition II dataset III [16]. The dataset was recorded from a female subject. The dataset was recorded at 128 Hz sampling frequency. It was band-pass filtered between 0.5 Hz and 30 Hz. The experiment included 280 trials of 9 s length. This is illustrated in Figure 2. Each trial starts with a fixed cross and an additional short audible warning tone. After a few seconds, a visual cue appeared, and the subject had to visualize the corresponding hand movements within 6 s.

### 2.2. Signal Preprocessing

When motor imagery EEG signal classification is performing, the raw EEG dataset is pre-processed to filter noise artifacts to obtain an expectation EEG signal. (1) Channel selection. The C3, Cz and C4 channels are the main acquisition channel for motor imagery EEG signals. To reduce the redundancy of the input signals, the noise, and the subsequent experimental design, we only use signals from channels C3 and C4. (2) Signal filtering. The first step is to remove the industrial frequency interference from the signal, which has been filtered out during the acquisition of EEG data. Based on the activation frequency of the motor imagery EEG signal, we use a band-pass filter of 8–30 Hz to obtain the frequency range bands that we need. It is worth noting that the filtering does not fully yield the desired frequency band. (3) Baseline correction. After filtering, baseline correction is applied to the signal to prevent the effect of data drift on the signal.

## 3. Methods

### 3.1. Enhanced Feature Differences

Some research has shown that when people perform left and right-hand motor imagery experiments [17], ERD/ERS patterns are observed occurring primarily in the corresponding sensorimotor cortex on both sides of the brain, namely in the C3 and C4 electrode regions. The results in [11] also show that the EEG signals generated through the C3 and C4 channels alone are feasible to classify the left and right-hand motor imagery tasks. When the Cz channel is introduced for classification, it does not improve the classification accuracy but introduces noise. Therefore, the EEG signals from channels C3 and C4 are chosen as input data in this paper.

Different people have different evaluation criteria for extracting useful features from a signal. To facilitate the automatic extraction of features of the signal by the classifier, we convert the EEG signal into a color time-frequency image using a wavelet transform. The images contain time domain information, frequency domain information, and the corresponding energy values of the signal. Then, the corresponding time-frequency images of the two channels are subtracted, relying on the logical symmetry of the C3 and C4 channels. Then, the feature-enhanced time-frequency images are fed to the classifier for automatic feature extraction.

A wavelet transform is an ideal tool for time-frequency analysis and processing for non-smooth, random EEG signals. The wavelet transform can fully highlight certain aspects of the problem, allowing for time subdivision at high frequencies and frequency subdivision at low frequencies. It can be automatically adapted to the requirements of time-frequency signal analysis and more complete information about the signal. The transformation equation that we use is shown in Equation (1):

(1) $$W_{x}(a,\tau )=\frac{1}{\sqrt{\left|a\right|}}\int_{-\infty }^{\infty }x\left(t\right)\psi^{*}(\frac{t-\tau }{a})dt,$$

where a is the scale factor, τ is x(t) the shift time of the mother wavelet. The factor a controls the scaling of the wavelet function, corresponding to the frequency domain information of the signal, τ controls the translation of the wavelet function, corresponding to the time domain information of the signal. In this way, the frequency component, and the corresponding position of the component in the time domain can be determined after the wavelet transform. ψ is the mother wavelet and we choose the morlet wavelet basis function.

**Figure 3:**
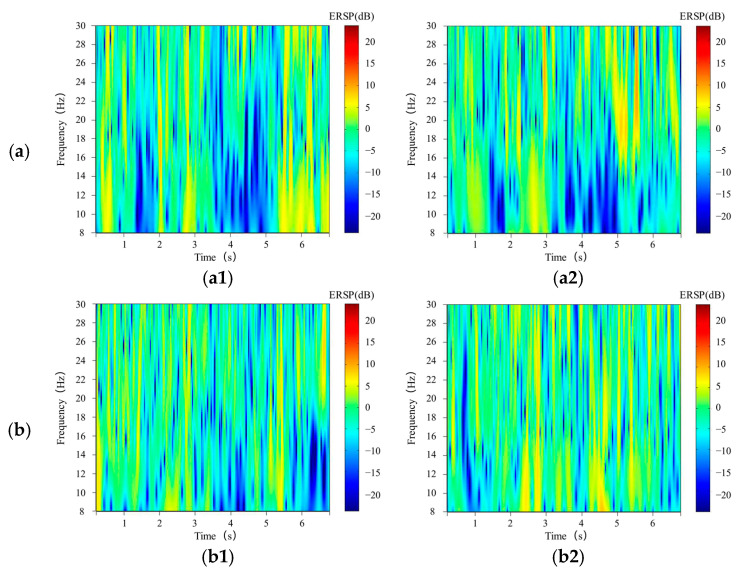
(**a**). Time-frequency images of the C3 (**a1**) and C4 (**a2**) channels when the subject performing the left-hand motor imagery. It can be seen that the energy of the C3 channel remains at a high level 4 s after the trail begins, while the C4 channel decreases significantly. (**b**). Time-frequency images of the C3 (**b1**) and C4 (**b2**) channels when the subject performing the right-hand motor imagery. Obviously, this phenomenon is opposite to that of Figure 3a.

The preprocessed MI-EEG signals are converted into two-dimensional time-frequency images by wavelet transform, as shown in Figure 3a,b. When hand movement imagery is performed, ERD/ERS phenomena occur in the cerebral cortex under the C3 and C4 electrode positions, with corresponding energy changes in the μ and β rhythms. Specifically, when left-handed movements are imagined, the energy in the corresponding frequency band of the cerebral cortex at the C3 electrode location rises and the energy in the corresponding frequency band of the cerebral cortex at the C4 electrode location falls. The temporal frequency images of channels C3 and C4 have opposite band-specific energy magnitudes, which were particularly evident in the μ rhythm section. Opposite energy fluctuates when right-handed motion imagery is performed.

**Figure 4:**
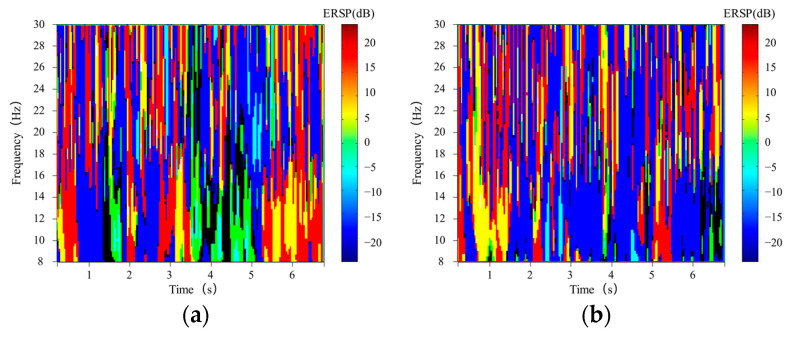
Final combined time-frequency images of left-hand (**a**) and right-hand (**b**) motor imagery. It can be seen that the energy difference between the two time-frequency images is obvious 4 s after the trail begins.

Depending on the image’s properties, different energies are represented in the image as pixel values of different sizes. When a classifier processes a time-frequency image, the classifier extracts the pixel values in the image as raw data for classification learning. Therefore, we enter raw data into the classifier to improve the accuracy of the classification. The basis for improvement is based on two main aspects: (1) C3 and C4 electrode positions are logically symmetrical about the longitudinal fissure of the brain; (2) Motor imagery has opposite energy changes in the occurrence areas of the left and right brain, which manifest as differences in pixel values on time-frequency images. To enhance the classifier accuracy by increasing this difference, we subtract the corresponding pixel values of the time-frequency images corresponding to the C3 and C4 channels. The image obtained by subtraction is used as input to the classifier. As shown in Figure 4, the input images of the two channels are combined and the feature differences are increased from the input side of the classifier.

### 3.2. Framework Construction

With the wavelet transform, we convert the original signal into a two-dimensional time-frequency image. We also fully extract the time domain, frequency domain, and the corresponding energy characteristics of the signal. We convert the problem of classification and recognition of signals into an image classification problem. Therefore, we design the classifier from the perspective of image classification. Based on the excellent performance of convolutional neural networks in the field of image classification, we use them as the base classifier and improve them. The classification accuracy is improved by improving the classification effectiveness of the classification model.

First, we build a basic convolutional neural network classification framework with two convolutional layers and pooling layers. To process the input data efficiently in the convolutional neural network, all input images are resized to a uniform size of 64 × 64. We select a convolutional kernel of a size 7 × 7. The convolution operation is a linear operation, whereas a neural network must fit a non-linear function. Therefore, we need to add activation functions, such as sigmoid function, tanh function, rectified linear unit (ReLU) function, etc. In this paper, we chose the ReLU function, which is defined and formulated with derivatives as shown in Equations (2) and (3):

(2) f(x)=max(0,x),

(3) $$f^{\prime }(x)=\left\{ \begin{array}{l} 1,x>0\\ 0,x\leq 0 \end{array}\right.,$$

When performing backhaul, we need to calculate the derivative of the activation function. As a result, the ReLU function is chosen, its derivative is always equal to 1 if the input is greater than zero. Compared to the sigmoid and tanh functions, the ReLU function overcomes the gradient disappearance, speeds up training, and has less computational overhead [11].

The convolutional neural network completes the descending and feature extraction of the input image by convolutional operations, but the dimensionality of the feature image is still extremely high. High dimensionality could lead to time-consuming calculations and over-fitting. Therefore, we introduce the pooling layers for downsampling to reduce data redundant information and simplify the complexity of the network. The area of the pooling layer is resized to 2 × 2 with a move step of 2. Then a dropout layer is added to reduce the model overfitting with the parameter 0.8. Finally, all features will be combined for classification via a fully connected layer. Finally, the optimizer selects Admax with a learning rate of 0.0003.

**Figure 5:**
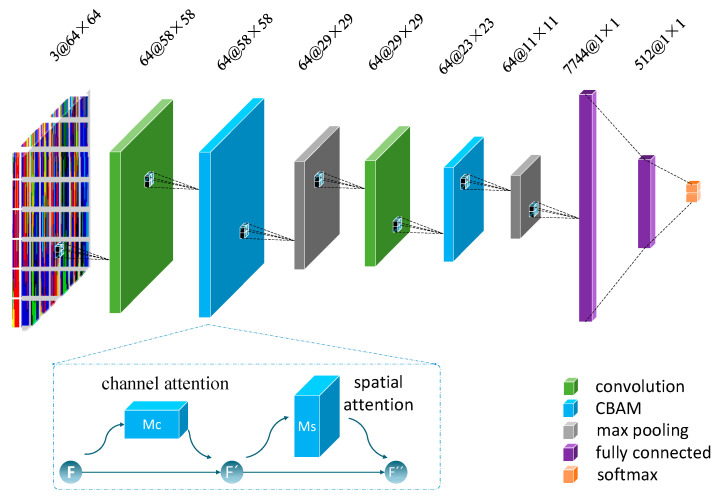
Proposed CNN structure with the CBAM module; the CBAM module is also described.

Due to the small dataset, we cannot rely solely on increasing the depth of the model to improve the accuracy of MI-EEG signal classification. Therefore, on the basis of considering the weight of the different channels of the input image and the spatial position of the image, CBAM is introduced into the convolutional neural network [18]. The attention module allows the deep neural network to focus on the parts that are most relevant to solving the problem at hand, rather than processing information from the entire input. The framework is shown in Figure 5. The module combines spatial and channel attention modules, considering both the importance of pixels in different channels and the importance of pixels in different positions in the same channel.

In IS-CBAM-CNN, the CBAM is embedded in the middle of the convolutional and pooling layers. The CBAM module includes two parts. The first part is the channel attention map $M_{C}\in {\mathbb{R}}^{C\times 1\times 1}$, which enables the selection of channels. The other part is the spatial attention map $M_{S}\in {\mathbb{R}}^{1\times H\times W}$, which selects the areas of the image space that require attention. C, H, and W are the indicators of the number of channels, height, and width of the feature map, respectively. As shown in Figure 5, the feature map output $F\in {\mathbb{R}}^{C\times H\times W}$ from the convolution layer passes through a channel attention module. The output $F^{\prime }$ is weighted and fed into a spatial attention module. The final weighting $F^{\prime \prime }$ is done to get the result to be passed to the pooling layer. The overall attention process can be summarized as shown in Equations (4) and (5):

(4) $$F^{\prime }=M_{C}(F)⊗F,$$

(5) $$F^{\prime \prime }=M_{S}(F^{\prime })⊗F^{\prime },$$

where ⊗ denotes element-wise multiplication.

The channel attention module focuses on which channels play a role in the final output classification result of the network. The feature map F is compressed in the spatial dimension by maximum pooling and average pooling to obtain two different descriptions of the spatial context: $F_{\mathrm{avg}}^{c}$ and $F_{\max}^{c}$. The two different spatial background descriptions are computed using a shared network consisting of a multi-layer perceptron (MLP) with hidden layers to obtain a channel attention map: $M_{C}\in {\mathbb{R}}^{C\times 1\times 1}$. The channel attention parameters are then obtained by summing after a fully connected layer, where both share the same fully connected network. The channel attention is computed as shown in Equations (6) and (7):

(6) $$M_{C}(F)=\sigma (MLP(AvgPool(F))+MLP(MaxPool(F))),$$

(7) $$M_{C}(F)=\sigma (W_{1}(W_{0}(F_{\mathrm{avg}}^{c}))+W_{1}(W_{0}(F_{\max}^{c}))),$$

where σ denotes the sigmoid function, $W_{0}$ and $W_{1}$ are the weights of the MLP.

The spatial attention module focuses on which locations play a role in the final output of the network. It improves the recognition accuracy and robustness of the model by reducing the interference of the background to the task. Two different descriptions (${\left(F^{\prime }\right)}_{\mathrm{avg}}^{s}$, ${\left(F^{\prime }\right)}_{\max}^{s}$) are obtained using maximum pooling and average pooling in the dimension of the channel. Two different descriptions (${\left(F^{\prime }\right)}_{\mathrm{avg}}^{s}$, ${\left(F^{\prime }\right)}_{\max}^{s}$) are combined and a spatial attention map ($M_{s}(F^{\prime })\in R^{\left(H\times W\right)}$) is generated using a convolution operation. The spatial attention is computed as shown in Equations (8) and (9):

(8) $$M_{S}(F^{\prime })=\sigma (f^{7\times 7}([AvgPool(F^{\prime });MaxPool(F^{\prime })])),$$

(9) $$M_{S}(F^{\prime })=\sigma (f^{7\times 7}([{\left(F^{\prime }\right)}_{\mathrm{avg}}^{s};{\left(F^{\prime }\right)}_{\max}^{s}])),$$

where σ denotes the sigmoid function, $f^{7\times 7}$ represents a convolution operation with the filter size of 7 × 7.

### 3.3. Evaluation Method

The improved framework is evaluated on the BCI Competition IV dataset 2b. There are nine subjects in this dataset. We test and evaluate the algorithm separately for each individual using kappa coefficients and accuracy rates. The kappa coefficient is a measure of classification accuracy. It represents the ratio of the model’s classification results to the reduction in error produced by a completely random classification, eliminating the effect of random classification accuracy. The kappa coefficient is defined as in Equation (10):

(10) $$kappa=\frac{p_{0}-p_{e}}{1-p_{e}},$$

where $p_{0}$ is the subject’s classification accuracy, $p_{e}$ is the assumed accuracy of the random classifier for the same data, and the value $p_{e}$ of the second classification is 0.5 [10].

There are nine subjects in the BCI Competition IV dataset 2b dataset. We took the first 2 sessions as the dataset for each subject, approximately 240 trails. As the performance of EEG experiments varied considerably between different subjects or for the same subject at different periods [19], we assessed the accuracy and kappa coefficient of the model using a 10 × 10-fold cross-validation method. With a smaller dataset, the 10 × 10-fold cross-validation makes full use of all the data, using fewer test data to obtain a higher level of reliability and eliminating the effect of within-subject variation on our results.

## 4. Results and Discussion

### 4.1. Results

To validate the performance of the framework, we compare it with SVM With Band Power Features (BP-SVM) [20], CNN’s With Stacked AEs (CNN-SAE) [10], Twin-SVM Method [21], Filter Bank CSP (FBCSP) [22] and Capsule Network (CapsNet) [14] using the BCI Competition IV dataset 2b dataset.

The results of the experiments are shown below. In Table 1, the accuracy of different frameworks is compared. It can be observed from the table that our proposed IS-CBAM-CNN framework performs better than BP-SVM, CNN-SAE, and CapsNet overall. Compared with BP-SVM, CNN-SAE and CapsNet, the average accuracy of the IS-CBAM-CNN framework improved by 9.4%, 2.0% and 1.2%, respectively.

For each subject, the accuracy of the IS-CBAM-CNN framework is higher than that of BP-SVM for all nine subjects. Except for the third and seventh subjects, whose accuracy is slightly lower than that of CNN-SAE, the IS-CBAM-CNN framework also shows excellent performance in the test validation for the remaining seven subjects. Among the nine subjects, the highest accuracy rate of CapsNet is 40.0% higher than the lowest accuracy rate, while the difference of IS-CBAM-CNN is 27.7%. The IS-CBAM-CNN shows a more stable classification performance.

For the mean standard deviation of the model accuracy, the mean standard deviation of BP-SVM, CNN-SAE, and IS-CBAM-CNN are 5.8%, 2.1%, and 1.8%, respectively. Compared with the other two methods, IS-CBAM-CNN shows good robustness.

In Table 2, the kappa coefficients of the different models are compared. Compared with Twin-SVM, FBCSP, and CNN-SAE, the average kappa values of the proposed IS-CBAM-CNN framework are improved by 9.0%, 6.6%, and 4.5%, respectively. The overall performance of the IS-CBAM-CNN method has improved. For each subject, six of the nine subjects outperform the remaining three models.

**Figure 6:**
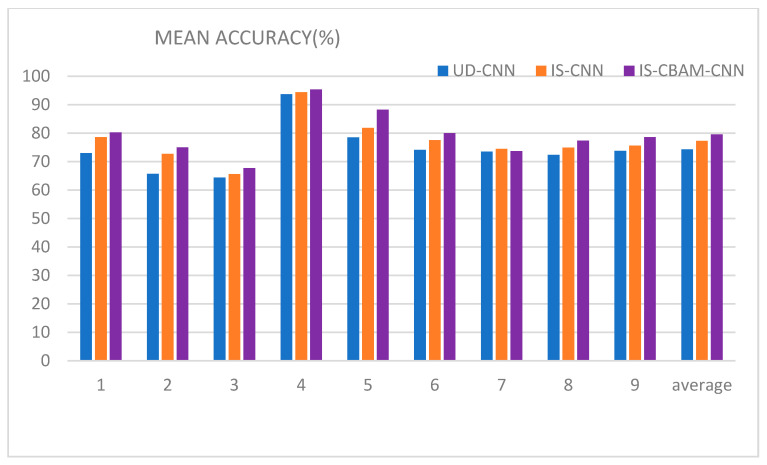
Comparison of the accuracy results of UD-CNN, IS-CNN, and IS-CBAM-CNN.

To verify the performance and advantages of the IS and CBAM modules, comparative verification experiments are carried out by replacing and removing the modules. The IS and CBAM are first removed on the basis of the IS-CBAM-CNN framework, and the input time-frequency images are stitched up and down as the input to the classifier, which we refer to as the up and down CNN (UD-CNN). Removing CBAM from the IS-CBAM-CNN framework, we refer to this method as IS-CNN. The three models are compared finally, as shown in Figure 6.

Figure 6 shows a bar chart comparing the accuracy of each subject under each of the three different methods tested. The average accuracy of the UD-CNN, IS-CNN, and IS-CBAM-CNN methods are 74.3%, 77.3%, and 79.6% respectively. The graphs more clearly represent the performance of the three methods, and for most subjects, there is some degradation in the performance of the method when either IS or CBAM is removed or replaced.

To evaluate our methods on another dataset, we use the same networks described before to classify data from BCI Competition II dataset III. Networks are trained with 140 trials in the training set and tested on 140 trials in the test set. As shown in Table 3, the accuracy of the IS-CBAM-CNN model is 90.7% and the accuracy of the competition winner’s algorithm is 89.3% [23], which is better than the winner algorithm of the competition. We also compare with a recent study [24] and the CNN-SAE algorithm. The accuracy rates of the study and CNN-SAE are 88.2% and 90.0%, respectively. The results of both methods perform lower than our proposed model.

### 4.2. Discussion

Through an extensive comparative analysis of experimental results, we confirm the feasibility of an approach that relies on the subtraction of image pixel values to increase feature differences, and then verify the effectiveness of the CBAM module in improving classification accuracy. Compared with prior MI-EEG classification methods, the proposed method shows superior performance in two aspects.

First, wavelet transform and time-frequency image subtraction (IS) are used to enhance the characteristics of different signals. Using time-frequency images as classifier inputs simplifies the feature extraction process for MI-EEG signal classification. However, because EEG signals are typically obtained using multiple electrode channels, how the time-frequency images are combined across multiple channels can have a significant impact on the accuracy of the final classification. Many research papers have proved that the C3 and C4 channels are sufficient to provide classification information for MI-EEG signal classification experiments. And the Cz channel could introduce noise interference in addition to providing little useful information, so we drop the Cz channel. Although a small amount of useful new information is lost, the introduction of noisy signals is also avoided. Relying on the logical positional symmetry of the C3 and C4 channels and ERD/ERS, we amplify the signal features by image processing to obtain more distinctive features to improve the accuracy of the classifier. In Figure 6, the effectiveness of the method for improving the classification performance is also verified by comparing UD-CNN and IS-CNN. Compared with previous time-frequency analysis, it has wide applicability while improving the classification effect. Specifically, more methods of generating time-frequency images can be tried in this framework, not only wavelet transform, such as short-time Fourier transform, and Hilbert–Huang Transform and so on. It provides more possibilities for improving classification performance. It is worth noting that image subtraction is both a strength and a limitation of ours (the necessary condition for the use of image subtraction in this paper is the logical symmetry of the C3 and C4 channels).

Second, the overall processing performance is further improved by the CBAM module, and a classification solution is provided for motor imagery EEG signals. The MI-EEG signal generates in specific frequency segments and fluctuating intervals, and the time-frequency images contain large areas of noise in addition to presenting pure MI-EEG information. The attention module is introduced into the convolutional neural network based on the temporal location and frequency distribution characteristics of the MI-EEG signal occurrence. By learning the channel information of the time-frequency images and the spatial location information of the different channel images, the weights of the different channel information and the different spatial location information are determined to improve the accuracy of the classifier. In Figure 6, the results of the IS-CNN and IS-CBAM-CNN comparisons also demonstrate the feasibility of the attention module.

In this paper, we start from the data feature processing and classifier model of motion imagery EEG signals and convert the signal processing problem into an image processing problem. Then we increase the feature differences, simplify the feature extraction process, introduce the attention module, and design the classifier from the perspective of image classification to enhance better results of signal classification.

## 5. Conclusions

This paper proposed a deep learning framework for MI-EEG classification from the perspective of image processing. The performance of the framework was evaluated on the BCI competition IV dataset 2b. The framework was improved in terms of both the input data and the classifier. First, we converted the signal into time-frequency images. Then, the IS method was used to synthesize the input and amplify the difference in energy characteristics at the level of the input. At this point, the signal recognition was converted to an image classification problem. Finally, relying on the convolutional neural network framework, which performs well in image processing, this paper introduced a CBAM module to reasonably extract spatial and channel information in order to improve the recognition capability and robustness of the framework. We validated the feasibility of proposed approach and compared it with other state-of-the-art methods. The experimental results demonstrated that the classification accuracy of the proposed method was better than the classical methods and state-of-the-art CNN-based methods.

There are still many continuous challenges and meaningful research directions that inspire us to keep moving forward. First, which time-frequency image generation method is more suitable for our proposed framework? We will further improve the quality of time-frequency images through different methods, such as short-time Fourier transform, Hilbert–Huang Transform, and other advanced methods. Second, the deep learning module still has limitations, including layer selection and network structure optimization. We will try more models and make reasonable parameter choices.

## Figures and Tables

**Table 1:** Competition IV dataset 2b accuracy (%) results for CapsNet, BP-SVM, CNN-SAE and IS-CBAM-CNN.

Subjects	CapsNet	BP-SVM	CNN-SAE	IS-CBAM-CNN
1	78.8	65.4 ± 4.7	76.0 ± 2.7	80.3 ± 1.5
2	55.7	58.5 ± 4.3	65.8 ± 1.9	75.0 ± 1.8
3	55.0	64.4 ± 5.9	75.3 ± 1.8	67.7 ± 2.6
4	95.9	92.7 ± 4.6	95.3 ± 0.4	95.4 ± 0.6
5	83.1	77.1 ± 6.6	83.0 ± 1.4	88.3 ± 1.5
6	83.4	71.4 ± 6.8	79.5 ± 2.5	80.0 ± 1.7
7	75.6	68.4 ± 7.6	74.5 ± 1.8	73.7 ± 2.2
8	91.2	68.8 ± 5.9	75.3 ± 2.6	77.4 ± 2.0
9	87.1	65.9 ± 6.1	73.3 ± 3.6	78.6 ± 2.1
Average	78.4	70.2 ± 5.8	77.6 ± 2.1	79.6 ± 1.8

**Table 2:** Competition IV dataset 2b kappa coefficients results for Twin-SVM, FBCSP, CNN-SAE and IS-CBAM-CNN.

Subjects	Twin-SVM	FBCSP	CNN-SAE	IS-CBAM-CNN
1	0.494	0.546 ± 0.017	0.517 ± 0.095	0.606 ± 0.030
2	0.416	0.208 ± 0.028	0.324 ± 0.065	0.500 ± 0.036
3	0.322	0.244 ± 0.023	0.494 ± 0.084	0.354 ± 0.052
4	0.897	0.888 ± 0.003	0.905 ± 0.017	0.908 ± 0.012
5	0.722	0.692 ± 0.005	0.655 ± 0.060	0.766 ± 0.030
6	0.405	0.534 ± 0.012	0.579 ± 0.099	0.600 ± 0.034
7	0.466	0.409 ± 0.013	0.488 ± 0.065	0.474 ± 0.044
8	0.477	0.413 ± 0.013	0.494 ± 0.106	0.548 ± 0.040
9	0.503	0.583 ± 0.010	0.463 ± 0.152	0.572 ± 0.042
Average	0.526	0.502 ± 0.014	0.547 ± 0.083	0.592 ± 0.036

**Table 3:** Competition II dataset III accuracies (%) results for the winner algorithm [23], deep network [24], CNN-SAE and IS-CBAM-CNN.

Subjects	[23]	[24]	CNN-SAE	IS-CBAM-CNN
Accuracy (%)	89.3	88.2	90.0	90.7
Kappa	0.783	0.764	0.800	0.814

## Data Availability

The databases used in this study are public and can be found at the following links: BCI Competition IV dataset 2b http://www.bbci.de/competition/iv/ (accessed on 16 May 2021), BCI Competition II dataset III http://www.bbci.de/competition/ii/#datasets (accessed on 16 May 2021).
